# Analectic

**Published:** 1832

**Authors:** 


### MISCELLANEOUS INTELLIGENCE.

### ANALECTIC, ANALYTICAL, AND ORIGINAL.

### I. ANALECTIC.

1. *Uterine Irritation.—The Morbid Conditions of the General System and of Particular Organs which result from the Continuance of Uterine Irritation.* By Dr. Addison, Guy's Hospital, London.—These of course, vary very much both in kind and degree, but chiefly in degree, according to differences in particular constitutions, and according to the susceptibility of individual organs.

The first complaint usually made by a female suffering from this irritation, is, of feeling *nervous*, probably with a disposition to be low-spirited; but if, in a well-marked case of the kind, you proceed to feel the pulse, and especially if it be your first visit, the chance is that you will perceive a distinct tremor of the hand, and remarkable acceleration and sharpness of the pulse, evidently arising from mental agitation—and a degree of mental agitation so great, in a naturally susceptible female, that if you attempt to soothe or encourage her, she will begin to sob, her lips quiver, and she bursts into a flood of tears. Even after she has sufficiently recovered her self-command, she experiences considerable difficulty in describing her feelings and sensations, and often appears to despair of satisfactorily communicating to you the nature of her ailment. She tells you, that, without any assignable cause, she gradually declined in health and spirits; that she has lost her wonted alacrity, has become indolent, and is easily fatigued by comparatively slight exertion; that she is readily flurried; that her heart often beats, flutters, or palpitates; that the impressions made upon her mind are altogether disproportionate to the causes producing them; that she is very prone to weep, and occasionally experiences sudden and transitory feelings of alarm and dread, especially during the night, without being able satisfactorily to account for them; in short, that both body and mind are in a morbidly sensitive condition, whilst general distress is strikingly depicted in her pale or dejected countenance. If you proceed in your inquiries to ascertain the state of the uterus, you are perhaps informed that she is regular; but if interrogated more narrowly, it will almost uniformly be found, that she suffers pain either before or during the flow, in the back or loins, and that in the intervals, she is troubled with leucorrhœal discharge. To these inquiries, she gives a reluctant reply; will often, perhaps from delicacy, conceal truth, or, if she acknowledge it, will probably add, "Oh, that is of no consequence; that is not my complaint; I have long been accustomed to that, and it does no harm;" and then winds up the case with telling you that she has taken a load of tonic medicines without benefit. If you ask her whether such questions were ever put to her before, you are generally answered in the negative. Such a case as this, with slight modifications, is of common occurrence, yet it most frequently happens, that, with the general disorder, we have decided derangement of some internal organ or organs; and of these, the organs of digestion appear to be almost uniformly the first to participate; indeed, the derangement of the digestive organs, to a greater or less extent, is so commonly associated with the general affection I have described, that one cannot but conclude that the general affection is most materially influenced, if not in part produced by it. It is sufficient, however, for our purpose to know, that the exalted susceptibility of the general system and this deranged condition of the first passages, are very commonly co-existent, however, they may stand in the relation of cause and effect.

The first appreciable disturbance of the stomach is most frequently a tendency to *flatulency*, which flatulency is productive of different effects in different individuals, although, in all, the stomach itself appears to be in a morbidly irritable condition, so as greatly to modify or aggravate the consequences that would otherwise arise from the presence of such flatulency. The patient experiences uneasiness at the scrobiculus cordis; she complains of a sense of load or distension after meals, or, if the stomach be uncharged with food, of prickings and anomolous pains in the organ, all of which symptoms are pretty uniformly relieved for a time by the expulsion of flatus from the stomach. In other cases, the irritation produced by the flatus about the cardiac orifice, excites a sympathetic affection in the throat, a sort of globus hystericus, which is variously described by patients, some calling it spasm, whilst others compare it to a mechanical obstruction, and indeed, one lady somewhat fancifully compared it to a bullock in her throat. It is a sensation, however, which will often last, in a greater or less degree, for days, or even weeks, with little intermission.

At other times the patient suffers from repeated vomiting, or is perhaps, seized suddenly, but only occasionally, with vomiting, preceded, accompanied, or followed by an irregularly inverted action, chiefly of the œsophagus, and attended with an ascent of flatus, so as, in some instances, to threaten suffocation.

Such are the ordinary affections of the stomach met with in the disorder to which I have alluded; affections, however, varying both in kind and degree in different individuals; affections, too, in which the whole alimentary canal appears more or less to participate, the patient being very commonly troubled with rumblings, distension and anomalous transitory twitchings in the bowels, symptoms undoubtedly depending upon the flatus and other contents irritating these morbidly sensitive organs. In more rare cases there appears to be a sort of inverted action of a greater or less portion of the alimentary canal; an inverted action commencing in some part of the bowels; an inverted action accompanied by a rumbling noise in these organs; an inverted action extending to the stomach, passing up the œsophagus to the pharynx, where it produces a most distressing sense of suffocation, and lastly communicating with and involving in one universal disturbance the brain and entire nervous system. This mysterious communication, so closely allied to the aura epileptica, is in all probability somewhat of the same nature, and may, perhaps, result from this same irritation in the alimentary canal being conveyed through the pneumo-gastric nerve to the brain; for when this takes place, the breathing is usually affected at the same time in a remarkable manner;—the whole assemblage of symptoms, the aberration of mind and bodily contortions constituting, when taken collectively, what in common language is understood by a hysteric paroxysm. There are, however, divers modifications met with—the patient being sometimes seized with violent and involuntary fits of laughter or crying, during the paroxysm, at other times she will lie in a perfectly motionless and insensible state, but with a natural pulse, for hours together, or she shall suffer from a more or less perfect paroxysm without its being preceded by any globulus whatever.

Hitherto, then, we have traced the effects of uterine irritation, as they appear in the form of a general morbid sensibility and mobility of body and mind, with flatulency and irritability of the stomach and bowels; which symptoms alone, but in different degrees, may continue to harass such patients for months, or even years, without either a well developed hysterical paroxysm or any other inconvenience deserving particular notice. But it remains to be observed, that the exalted state of the nervous system is not, in a considerable proportion of cases, evinced solely by morbid susceptibility and excessive or irregular action, but, moreover, by *morbid sensation*, affecting different parts or organs of the body, and this too, varying from the slightest uneasiness to the most exquisite torture.

Now, it is to these painful affections that I am anxious to direct your earnest and special attention; because, in the first place, it is to relieve these that you will most frequently be called upon to render your assistance, and because, in the next place, these painful affections are perpetually misunderstood and mistaken, to the serious detriment of the patient. I repeat, then, I am anxious, most anxious, to impress upon your minds the nature of these secondary painful affections, their most frequent seat, and the various sources of fallacy by which you may be betrayed into error.

Of these painful affections, the most serious, or at least the most prominent, and certainly by far the most interesting, are those which attack the abdominal viscera, as these are repeatedly mistaken for inflammation, and treated accordingly. I shall not stop to inquire why those viscera, supplied by the ganglionic system in general, or why the abdominal viscera in particular, should be more liable to suffer such painful affections than other parts of the body supplied by the ordinary nerves of sensation and voluntary motion; suffice it to say that such is the fact.

Of the painful affections of the abdominal viscera, the most frequent are,

1st. A pain seated under the left mamma, or under the margin of the ribs of the same side.

2d. A pain under the margin of the ribs of the right side.

3d. Pain in the course of the descending colon.

4th. Pain in the course of the ascending colon, especially towards the right hypochondrium.

5. Pain affecting the abdomen generally.

6th. Pain in the region of the stomach.

And lastly, pain in the region of the kidneys, sometimes extending down the course of the uterus to the bladder.

Such are the painful affections usually met with, attacking the abdominal viscera; affections, too, which, in point of frequency of occurrence, observe the order in which I have enumerated them. You will have noticed that I am reserved in referring these pains to any particular organ, contenting myself with the *fact* that the pain is felt in a particular *situation.* The truth is, considerable difficulty presents itself in ascertaining positively what particular organ is affected, as the natural functions of the part or organ do not, in such cases, appear to be necessarily disordered, or at least to such an extent as to indicate with certainty that it is the seat of the pain complained of by the patient; neither can we have the usual aid of *post-mortem* examinations, as few die of such complaints whilst, if the patient be cut off by another disease, no organic lesion is discoverable to satisfy our inquiries. Occasionally, however, this question may be decided during life, as we shall see presently.

With respect to the *pain under the mamma, or under the margin of the ribs of the left side,* this is out of all proportion of most frequent occurrence, and will often last for weeks, or even months together, with but little intermission. This pain is very circumscribed; it is not necessarily or constantly increased by a deep inspiration or by external pressure, although this is occasionally observed; it is seldom attended with cough; it is not materially affected either by a charged or by an empty state of the stomach, but varies in its intensity, and now and then goes off altogether for a few minutes, hours, or even days, or the pain shall subside, and be succeeded by a mere uneasiness or sense of fulness in the part. This pain, as I have said, is of extremely frequent occurrence, and is very often associated with palpitation of the heart, or, what is much more usual, with unnatural pulsation of the organ, if I may be allowed the expression; *i. e.*, the patient is conscious of the heart's action, or she feels as if its impulse were communicated to a part so sensitive as to excite distinct sensation, which, you know, is not the case in a state of health. With respect to the precise source of the pain, I confess myself at a loss to speak with confidence or certainty, but am upon the whole inclined to assign it, when complained of under the left mamma, to the cardiac orifice of the stomach; or at least in one case in which it had prevailed for a considerable period, and in a very aggravated degree, I was led to this conclusion. The young woman, to whose case I allude, died suddenly in a fit, and I examined the colon, spleen, heart, and stomach, with the minutest attention, when the only indication of irritation I could detect was a ring of very delicate vessels, or rather a blush of redness, surrounding the cardiac orifice of the stomach, such as might be supposed to be the result of any continued irritation or spasmodic action. Whatever may be its precise seat, it is repeatedly but erroneously, supposed to be purely of an inflammatory nature, and consequently is mistaken for and treated as pleuritis or splenitis.

The second painful affection to be noticed is that *seated close to the margin of the ribs on the right side.* This pain, although occasionally circumscribed almost to a point, usually extends from the scrobiculus cordis along the margin of the ribs, nearly to the loin of the side affected; it is neither considerably nor uniformly increased by a full inspiration, yet this is occasionally observed. External pressure, however, aggravates the pain, and sometimes in a very remarkable degree, whilst, in some instances there is such tenderness, that the patient shrinks from the slightest touch. The pain now and then shoots through to the back or between the shoulder-blades, but very rarely to the top of the right shoulder. This pain will occasionally remain, with slight remissions, for weeks or even months; at other times, it subsides altogether, and is succeeded, like the pain under the left breast, by a sense of fulness or tension of the part. As to the actual seat of this pain, I again confess myself incompetent to decide. I have sometimes supposed it to be in the *colon,* as it may now and then be traced from the margin of the ribs into the right iliac region; in other instances, I have supposed it to be seated in the *duodenum,* from its being occasionally attended with sickness, from its being aggravated during the operation of mercurial purgatives, and from its being in some rare cases attended with a remarkable sallowness or icteritious aspect of the countenance, and indeed with almost complete jaundice.

Here again, then, I must leave you in doubt, merely remarking, that it is not inflammatory, although repeatedly mistaken for hepatitis, and treated as such.

The next painful affection to be noticed is *that seated in the course of the descending colon.* This is not unfrequently associated with the pain under the left mamma, but is also observed to exist alone, extending from below the ribs to the *sigmoid flexure of the colon.* This, like the others described, is variable in its degree, and although more or less permanent, sometimes remits for hours, days, or even weeks together, and again returns. It is, however, more decidedly and obviously affected by flatulence, than the pains in the other situations mentioned; thereby more clearly pointing out the seat of the malady. The movement of the flatus is occasionally attended with a gurgling or rumbling noise, and a simultaneous aggravation of the local pain in the part. This is now and then mistaken for colitis, or for some organic lesion of the organ.

Fourthly, *Pain in the course of the ascending colon.* I have had occasion to observe, that the pain already described as situated behind the margin of the ribs on the right side, sometimes extends down the course of the colon as far as the iliac region; it not unfrequently happens, however, that the pain is felt exclusively in the situation of the ascending colon, and like that on the opposite side, varies in degree at different times, or for a period disappears altogether. This pain too, after a time, is attended with considerable tenderness, so that the least pressure creates inconvenience.

Fifthly, *Pain affecting the abdomen generally.* This is by no means of rare occurrence, and in some instances so closely resembles general peritonitis as to be mistaken for, and treated as that complaint. Indeed, I know of no disease more puzzling than this, and it was not, I confess, till I had witnessed several such cases, and attended more minutely to the history and progress of the disorder, that I became convinced of its true nature. It may be called a general *neuralgia* of the abdomen. It is sometimes attended with a tympanitic, and at other times with a flaccid state of the bowels, the former being by far the most distressing. The pain is complained of over the whole of the belly, and the slightest touch, in many instances, cannot be borne, such is the extreme sensibility and tenderness of the parts.

If you watch the case attentively, you will, in general, soon detect some incongruity in the symptoms, to excite doubt and suspicion; but yet, so close is the resemblance in some cases, as almost to set positive diagnosis at defiance. A case of this kind lately occurred to me, where I was in so much doubt, that, to err on the right side, I treated it as peritonitis, although the history of the patient, and the condition of the uterus, told against it: I soon became convinced that I had been wrong, as I shall presently explain to you. It may be observed, too, that this general pain of the belly, like peritonitis, frequently occasions but little distress, unless pressure be applied, and like peritonitis also, it suffers an aggravation at intervals, an aggravation apparently in both cases depending upon the spasmodic action or griping in the bowels.

The *sixth painful affection* is that attacking the *stomach* in particular. This pain is for the most part strongly marked, and the more intense the disorder, the more positive is the evidence of its being really seated in the organ mentioned. Thus it will sometimes come on suddenly, occasioning the most excruciating agony; the patient screams from the violence of her sufferings, her countenance is expressive of the greatest distress, and she leans forward or bends the body in order to diminish the pressure of the abdominal parietes; or, she says that the pain is drawing her double. This, in some cases, will last with little mitigation for several minutes, or even hours, the patient, the whole of the time making loud complaints, and declaring that she must die if not speedily relieved. This pain will probably remit, and be succeeded by another much less severe, though more permanent; both the intense and more moderate pain being much increased by pressure made upon the epigastrium.

*Lastly.*—Such patients occasionally experience a sudden and severe attack of pain in the region of the kidneys, to which region it may be exclusively confined, till it disappear altogether, or it shall extend from thence down the course of the ureters to the bladder, or the bladder alone shall be affected with pain; in either of the latter cases the patient generally experiences more or less dysuria.

Having pointed out the ordinary local affections arising from or connected with uterine irritation, I need only add, that you will often in the same individual meet with more than one of them existing at the same time; or, what is more usual, you will have them alternating with each other; whilst you will also observe the greatest variety in the relative severity of the local and general disorders mentioned; the general derangement prevailing occasionally in a very high degree with but little local pain, and *vice versa.*

*Treatment of Uterine Irritation.*—In the treatment of this disease, the *indications* are, 1st. To correct the morbid condition of the the uterus. 2. To remove or mitigate the violence of troublesome symptoms in any individual case; and 3d. To restore tone and vigor to the general constitution.

In thus laying down the indications of cure, you will perceive that I have ventured to reverse, in some measure, the ordinary mode of procedure. I repeat to you, that the condition of the uterus, to the continuance of which I ascribe the various disorders enumerated in this lecture, has in most instances been altogether overlooked, or at least, if observed, it has only been in these cases which have had some of the characteristic symptoms present, in a strongly marked degree, such as excessive or very painful menstruation, or profuse leucorrhœal discharge; and even then the condition of the uterus has been usually looked upon as a mere effect or consequence of the nervous or hysterical state of the entire frame, or in other words, it has been considered as merely participating in a universally morbid sensibility. Hence, the primary indication has commonly been to restore strength to the general system, regardless of the local disorder, although the ill success of the treatment founded thereon, has at all times been frankly acknowledged; so much so indeed, as almost to have placed the disorders in question amongst the *approbria medicinæ.* Having been led however, to take a different view of the subject, I am necessarily induced to make the correction of the state of the uterus the first, or at least the principal indication, although of course the second indication, and that of allaying troublesome symptoms, must often go hand in hand with, or in aggravated cases, even take precedence of it. What then are the means best calculated to remove the morbid condition of the uterus, the irritable state of the organ? In the cases in which the uterine disorder has attracted attention, it has been recommended to bleed generally, to take blood by cupping from the loins, or by leeches from the region of the pubes, or from the pudendum; to purge, to give anodynes, to employ the warm bath, and to keep the patient in a recumbent position, even for months at a time. Of this kind are the remedies recommended in an excellent work recently published by a distinguished member of our profession—I mean Dr. Gooch. That gentleman treats of the complaint as it is attended with actual tenderness of the os uteri to the touch, when an examination is made *per vaginam.* He enumerates the remedies I have mentioned, but with these expressions of extreme distrust which mark the man of candor and truth, and with that modesty which, when united to extensive experience, commands the assent of every reader. Now, that all these remedies may be occasionally serviceable, or even necessary, I am not presumptuous enough or inclined to deny: depletion, general or local, in the plethoric, and anodynes and laxatives in most cases; yet, speaking from experience, this object, this grand object is secured with much greater certainty, and much more speedily by applications made directly to the uterus itself, and parts adjacent. The applications to which I allude, are *cold astringent washes,* injected *per vaginam* by means of a proper syringe. The ordinary womb syringe answers the purpose exceedingly well, but one of any convenient shape may be used, provided it be sufficiently large to contain from four to six or eight ounces of fluid. The injection should be introduced with such a degree of force as shall secure its application to the upper part of the vagina, and to the os uteri; and the operation should be repeated two, three, or four times a day, according to the circumstances of the individual case.

Either the mineral or vegetable astringents should be used; the former, however, I prefer, as they do not stain the patient's linen, and consequently are not so much objected to. With respect to the precautions to be observed in the employment of these injections, very few are required beyond what common sense would dictate, should the injection occasion smarting, which is by no means unfrequently the case at first, it may be diluted with water, or water alone may be used till the original tenderness subsides, which for the most part it will soon do. It will also be prudent to instruct the patient to relinquish it a little before the expected period of menstruation, and to resume it as soon as that period is over. These are almost the only precautions I have ever deemed it necessary to observe. Although in very irritable habits and especially when the stomach is liable to be effected with pain and spasm, it may be as well to direct the wash to be used tepid at first, gradually diminishing the warmth till it is brought to the ordinary temperature of the patient's apartment, which will pretty uniformly be borne exceedingly well after a time, except perhaps during a few of the coldest months in winter. The wash I most frequently employ is the *Liquor Aluminis Compositus,* of the London Pharmacopœia; that is, two drachms of *alum,* and two of *sulphate of zinc,* to a pint of water. This practice must be persevered in for a length of time, proportionate to the obstinacy of the case, and the effect it produces. Indeed, I myself recommend females never to relinquish it, but to employ it from time to time, as long as they continue to menstruate, to prevent the recurrence of the disorder, and its unhappy consequences. I have said that the patient should desist from the use of the injection, a little before and during the menstrual period; but she ought also to be specially cautioned against using any violent exertion, or undergoing any unusual fatigue at that time, as nothing so completely thwarts your purpose as imprudence committed whilst the irritable uterus is performing its functions.

When the uterine irritation is characterized by frequent and excessive flow of the menses, I have directed the patient to remain quiet in or on the bed, and to desist from the wash during each recurrence; not having ventured to carry the practice to the extent of attempting to restrain even such excessive discharge by local astringents. But when there is decidedly painful menstruation, or pain felt in the *womb, loins, or thighs*, before the appearance, although there be no leucorrhœal discharge whatever in the interval, I nevertheless apply the cold wash to such subjects, precisely in the same manner as if the leucorrhœal discharge were present, on the principal that this leucorrhœal discharge is itself a mere symptom or effect of the state of the uterus and neighboring parts, which I am anxious to remove. Such is the *local* treatment that experience and observation have led me to adopt, and which, after a long trial, I venture, with some confidence, to recommend to you. It is just possible, that circumstances not noticed by me may occasionally occur to interfere with or forbid the practice; but I speak of generalities, and not of universal principles—universal principles are unknown in our profession.

The second indication—*to allay or remove troublesome symptoms*—must, of course, be variously fulfilled, according to the circumstances of the particular case, but the remedies employed for the purpose will necessarily co-operate with the local treatment, to afford relief to the patient; unless, indeed the severity and character of the troublesome symptoms be such as to render the *immediate* application of the wash either doubtful or hazardous—as *e. g.* when the stomach is the seat of mere spasm, or the bladder or abdomen generally acutely painful, in which cases the second indication must take precedence of the first. Should the symptoms merely consist of the susceptible state of body and mind before described, or should this be accompanied by that modification of derangement of the digestive organs characterized by flatulency and irritability of the stomach and bowels, the general treatment need be very simple. I say nothing of *bloodletting*, as the propriety or impropriety of this must be apparent from the state of the circulation, always keeping in mind that such subjects bear bleeding badly, especially in large quantity, and that mere *frequency* even with *sharpness* of the pulse, is, in the highest degree, fallacious: a full, hard pulse, with considerable heat of skin, and more or less throbbing or pain in the head, will point out the expediency of a moderate bleeding. *Purgatives*, or rather *Laxatives*, ought never to be neglected, but much caution and judgment are required in their proper selection and application. The bowels, it is true, are generally costive, but it must be recollected that they are as uniformly in a weak and irritable state; hence those purgatives should be selected which give the least pain, and have the least tendency still further to weaken their tone. Watery saline purgatives are ill suited to the majority of such cases; they often perform their office imperfectly, and although they may not produce much pain, which they often do in such subjects, they nevertheless tend, by frequent use, further to impair the tone of the bowels, and to increase the disposition to flatulency. Except, therefore, that the commencement in plethoric subjects, or where the disorder exists in connexion with menorrhagia, the saline purgatives are not commendable. *Castor oil*, when it agrees with the patient's stomach, will be found more generally serviceable, perhaps, than any other. In many cases, the warm and resinous purgatives, though at all times more irritating, answer exceedingly well, emptying the bowels of their fæculent contents, and expelling flatus without leaving any increased tendency to flatulency afterwards, as is the case with the watery saline purges. A little *Compound Extract of Colocynth*, or equal parts of this and *Extract of Rhubarb* with a little *Extract of Hyoscyamus* to obviate griping; or the above extracts may be given with a little *Blue pill*, should a mercurial laxative be indicated by the appearance of the secretions. Calomel very often gripes severely, whether alone or combined; yet such is the variety of constitutions met with, that the compound extract of Colocynth with Calomel, often proves the most effectual and not the most painful purge. One or other, then, of these laxatives may be necessary once or twice a week.

In the case that I am now treating of, that is, where there is no abdominal pain, the medicine that I have found to afford the greatest relief is unquestionably the *Ammonia*, given either in common mint or camphor Julep, alone or with a few grains of *Magnesia;* about ten or fifteen minims of the *Liquor Ammoniæ Subcarbonatis*, with from eight to ten or fifteen grains of *Subcarbonate of Magnesia* two or three times a day. If it create pain in the stomach, or a disagreeable sense of heat there, these effects may sometimes be obviated by adding about half a drachm of Tincture of Hyoscyamus, a drachm or so of the Tincture of Hops, to each dose. This ammoniacal mixture seems, by its stimulus, to expel flatus, and thereby afford relief to many of the uneasy and unhappy sensations, and to raise the spirits of the patient. I have often given the Ammonia along with the Mistura Myrrhæ of Guy's Hospital, an ounce or a dose of which contains, I believe, about twenty grains of Myrrh, and is made with the Decoction of Liquorice root.

But suppose that, with more or less of the general symptoms, we have pain under the left mamma, or in any other of the situations pointed out, what modification of treatment ought we then to adopt?

The pain under the left mamma is of such frequent occurrence, that it becomes a matter of the very first practical importance to bear it carefully in mind when called upon to treat the disorders of young females. There is no local pain more frequently mistaken, and there is, perhaps, no local disorder so maltreated as this. Over and over again have I known patients blooded, cupped, blistered, and anointed with acrid ointments, for months in succession, not only without relief, but with the most serious injury to their general health, whilst the uterine irritation has been altogether overlooked. Indeed, in thus declaring and denouncing such a mistake and such bad practice, I candidly confess that I am only declaring and denouncing "*quœque ipse miserrima vidi, et quorum pars magna fui.*" In truth, it was the frequent occurrence of this obstinate and intractable pain that first led me to a close and minute investigation of the subject of uterine irritation, having, like others, repeatedly blooded, cupped, and blistered in vain in such cases. Since, however, I have been guided in the treatment by the principles I have ventured to lay before you, my success has been much more certain and decided; and when the treatment founded thereon has not fully answered my expectation, it has at least been attended with the negative advantage of inflicting no unnecessary injury on the patient's general health.

In pointing out its non-inflammatory nature, do not imagine that I contend for the impropriety of bleeding or cupping or blistering in every instance. On the contrary, in plethoric subjects, one or two general or local bleedings may occasionally be of service; but merely, I believe, as such practice might be expected to afford relief in an ordinary colic, or any other similar painful affection. Depletion, however, is very far from being, in general, necessary, and probably will, in a majority of cases, be positively hurtful, teazing and exhausting the patient without adequate or permanent benefit. I have known Cupping, Leeches, a Blister, or an Opiate of Belladonna plaister, afford relief; but they all often fail, and will generally do so, or at least be merely followed by a temporary respite, unless the condition of the uterus and other circumstances I have pointed out, be attended to. Hot fomentations to the part will sometimes afford relief, but are attended with the same uncertainty as the other local remedies mentioned. Under the use of the injection, however, and the ammonial mixture with Tincture of Hyoscyamus, or three, four, or five grains of the Extract of Conium, in the form of pill, alone or with a grain or so of blue pill, according to the state of the secretions, the pain will generally yield, although the patient, as might be expected, will for a longer or shorter period be liable to a recurrence, until the original irritation, and its effects upon the abdominal viscera, shall have been overcome by a steady perseverence in the use of appropriate remedies. In some instances the ammonia cannot be borne, when the plain Camphor, or Mint julep, may be substituted, with half a drachm or so of the Tincture of Hyoscyamus, or four or five grains of the Extract of Conium, may be given in the form of pill twice or thrice a day. In other cases again, you may try six or eight drops of Batley's Liquor Opii Sedativus at bed time, unless it offend the stomach or the head. Should it constipate the bowels, proper laxatives should be had recourse to, in order to obviate such an effect; I prefer, myself, the Conium, or Hyosciamus; unless, however, it happen thus to disagree with the patient, the ammonia will answer best, guarded of course by the anodynes.

In those cases in which the pain shifts its seat from time to time, I

limit my *external* remedies generally, to hot anodyne fomentations; such as the decoction of Poppy-heads, or what often answers very well, the hot infusion of Chamomile Flowers, the flowers themselves being inclosed in folds of flannel, the more effectually to retain the warmth. I may also here observe again, that this tendency to shift its seat, together with the unsteadiness in the degree of the pain, form the most important diagnostic indications; whilst in most instances, we shall find, on inquiry, that the patient had suffered more or less from the complaint for some time before she applied for advice. Attention to these circumstances will materially assist us in our diagnosis, not only in these, but in the other painful or neuralgic affections attacking the abdominal viscera.

When the pain is situated in the *right hypochondrium*, or under the margin of the ribs of the right side, it is, as I have before said, repeatedly mistaken for, and treated as, hepatitis, and many are the times that I have known poor delicate women blooded, cupped, blistered, and salivated on this supposition, till they have been brought almost to the very brink of the grave. It is to be distinguished by the symptoms and circumstances already pointed out, and when ascertained, is to be treated upon precisely similar principles to those so minutely detailed. In these cases, patients sometimes tell us that our medicines, especially mercurial and resinous purgatives greatly aggravate the pain of the part during their operation. Cupping, Leeching, and blistering, will each occasionally, but only occasionally, afford relief; whilst, generally speaking, I would say that in this form of the complaint hot anodyne fomentations, assiduously applied, are more decidedly beneficial than in the former case, but in other respects the same means may be pursued. In some cases, four or five grains of Dover's powder, twice a day, will afford relief; in other instances, the more powerful combination of calomel and opium has been tried with effect. The calomel, however, ought to be given with great reserve, and never, if possible, to such an extent as to effect the system; in short, in all cases, the less violent measures we employ the better.

When the pain seizes the track of the colon on either side, the same treatment will apply, as it will also when the stomach becomes the seat of the pain; unless, indeed, severe spasm accompany it, when more active measures must be had recourse to, as a full dose of Liquor Opii Sedativus, or even a full dose of *Laudanum* and *Sulphuric Æther*, as I shall presently show.

I have said that the pain occasionally attacks the whole of the belly, exactly simulating acute peritonitis. This may be attended either with a flaccid or tympanitic state of the intestines, the latter proving by far the most painful of the two, the slightest touch causing the patient to cry out.

This generally diffused pain is not unfrequently associated with that modification of uterine irritation marked by excessive menstruation, both in point of quantity and frequency. But in such cases, whether with or without menorrhagia, it will require all your tact and discernment, and what is more, it will require all your philosophy and forbearance, to abstain from copious depletion under an apprehension that it may be peritonitis. The history of the case will generally raise a doubt, and will often bring conviction; but if a doubt do really exist, it will always be prudent to err or the right side, and treat it as inflammation.

When *unaccompanied* by excessive menstruation, the same general rules of treatment are to be observed as have already been detailed; moderate bleeding if much plethora and headache, with a full pulse, prevail; and the free use of laxatives and fomentations. In both forms of the complaint, to relieve the pain in this situation, I hold the Liquor Opii Sedativus to be by far the most efficient remedy, as well as attended with the least inconvenience to the patient. With due attention to the bowels, therefore, six, eight, or ten drops may be given, every night, or every night and morning, according to the urgency of the pain.

*When attended with excessive menstruation*, there very usually prevails a plethoric condition of such irritable subjects, so that a moderate bleeding will commonly form a very good mode of commencing our practice: or if there be much forcing or bearing down, the patient may be cupped from the loins. In all cases of this kind, too, the patient, during the flow, and for a short time before the expected recurrence, should be strictly enjoined to remain in the horizontal posture; to be kept cool, and live upon low and bland diet. Nothing so certainly, or so greatly aggravates cases of menorrhagia, even when without obdominal neuralgia, as the ill-founded and ill-judged practice of giving tonics and stimulants on the erroneous supposition that such excessive discharges are the result of weakness. Repeatedly have I known the most serious mischief result from the practice here pointed out; patients having been given Bark, Port Wine, and cold Porter, merely because they complained of great debility, and because such complaints of debility have tallied with the preconceived notion of the practitioner, that the disease had its origin in weakness. You must be careful to distinguish a *weak and relaxed*, from a merely *irritable*, or a *delicate* and *irritable* habit. It is the latter in which you most frequently meet with the menorrhagia, of which I now speak. The excessive discharge is intimately connected with such irritability of the system at large, and of the uterus in particular; tonics and stimulants only tend, therefore, to produce excitement; to augment the action going on in the irritable uterus, and thereby to increase the sufferings and exhaust the strength of the patient. As far as the discharge is concerned, therefore, your object is to allay the vascular excitement by omderate depletion and purging, and to allay irritation by anodynes and rest. A free action on the bowels exerts so powerful an influence over excessive uterine discharge, that I am often induced to administer divided doses, either of the Magnesia or Salts mixture, or of the Salts dissolved in the Compound Infusion of Roses; endeavoring to obviate the increase of the neuralgic pain of the belly, which is liable, when it prevails, to be aggravated by the Salts, by giving at the same time about five grains of Extract of Conium or extract of Hyosciamus, three times a day instead of the Liquor Opii Sedativus. In general, however, other laxatives may be given occasionally, to maintain a free discharge from the bowels, and to counteract the effect of opiates, should we determine upon a trial of them; for this purpose Castor oil, or Calomel and Rhubarb may be given; or the object may be, to a certain extent, accomplished by the use of glysters. If in spite of our depletion, laxatives, and rest, the flood still continues to prevail, without neuralgia of the abdomen, I generally direct the patient to apply cold vinegar and water, by means of a sponge, to the lower belly, two or three times a day; employing at the same time anodynes, or anodyne glysters, in order to allay the irritability of the womb itself.

If you have flooding alone, then, without neuralgia of the belly, you will bleed generally or locally, according to the state of the system at the time; keep up a free action on the bowels; keep your patient cool, and on low diet, and administer anodynes, either by the mouth or by glyster, with or without cold sponging, according to the severity of the case. Should flooding prevail along with the neuralgia of the belly, precisely the same remedies, with the exception perhaps of the cold sponging, will be required; whilst, if the neuralgia exist alone, the case becoming much more simple, must be treated on common principles; i. e. general bleeding, according to the degree of excitement of the heart and arteries; perhaps leeches to the belly: hot fomentations, laxatives and anodynes, either given by the mouth, such as Liquor Opii Sedativus, Conium, or Hyoscyamus; or in the form of glyster, as thirty or forty minims of Laudanum, with a little thin starch. Even in flooding cases I never give the lead, as I never fail by more gentle means to subdue the disorder, and I have known the lead induce severe colic.

I need only observe further, that if you should feel disposed to try the Oleum Terebinthinæ, I should consider it best suited to the general neuralgic state of the belly, without flooding or excessive menstruation in any form. From two drachms to half an ounce may be given with three or four drachms of castor oil. I am, however, averse to it myself, from its liability to offend the stomach and be rejected, but more especially from its tendency to act with violence on the kidneys. Such are the means which I have found most frequently of service in alleviating the more prominent and distressing symptoms attendant upon uterine irritation; means, I confess, often insufficient for the purpose, unless long persevered in, and this too in conjunction with the local treatment already mentioned. Many other questions and expedients will undoubtedly suggest themselves in particular cases, as Camphor, Musk, the Warm bath, and such like; or should the secondary local pains described not be considerable, the *fetid gums* especially *Asafœtida*, will often afford some, though tardy relief. Indeed these fetid gums have attained a higher character as *antihysterics.* bnt appear to me chiefly, if not exclusively, adapted to relieve some of the symptoms more particularly connected with the condition of the bowels;—they expel flatus, raise the spirits, and moreover appear to restore tone to the alimentary canal, provided the original source of the evil be attended to at the same time. In the less painful forms therefore of the disorder, these fetid gums are worth a trial, and will often afford considerable relief to the feelings of the patient.

Much of what has been said, merges in the *third indication;* viz. to restore strength and vigor to the general habit. Of the best means of accomplishing this I need say very little, beyond remarking, that the *early* use of *tonics* has been extensively adopted, and has consequently been given a fair trial, whilst almost all are agreed to the very unsatisfactory results, in a large majority of cases; the cause of which you will now be able, in some degree, to appreciate; I mean, of course, inattention to the local irritation, from which I suppose the whole mischief to proceed. In the highly irritable and susceptible state of the body at large, and of the alimentary canal in particular, the more stimulating or irritating tonics cannot be borne, creating sickness, pain or uneasiness at the stomach, loss of appetite, headache, and divers unhappy sensations not easily defined by the patient. Chalybeates not only offend in this way, but are extremely apt to excite the uterus, so as to produce excessive menstruation, if it did not previously exist, or to aggravate it, if it have already prevailed. Exceptions do undoubtedly occur, but the objections to them stated are founded on personal experience. The *Sulphate of Zinc*, however, may sometimes be given early, with advantage, provided it do not offend the stomach. A grain to begin with, may be given night and morning, alone, or with a few grains of Extract of Conium, or of Hyoscyamus, or with a little of the Pil. Galban. Comp.; or it may be given along with two or three grains of the *Extract of Gentian* or *Extract of Bark;* or these latter may be given without the Zinc. Any of these may be tried and persevered in, till the local disorder, and the most prominent of the general symptoms shall have been subdued to a certain extent, when less reserve may be observed as to the nature of the tonics we employ; the chief of them are *Bark, Bitters, Chalybeates*, the *Cold Bath, and Country air.—Boston Med. Journal.*

2. *M. Delpech on Varicocele.**—The subject of varicocele has been studied, with some attention in this country, especially by Sir Astley Cooper, who has communicated much useful information on its causes, nature, and treatment, in his splendid work on the diseases of the Testis, Yet still our remedial measures are imperfect, and though we may palliate, we can seldom cure. Variocele is frequently productive of much inconvenience to the patient. M. Delpech, for instance, asserts that he has known military officers obliged to abandon their profession in consequence. Varicocele also tends to occasion wasting of the testis, partly, we suppose, from the pressure exercised upon the organ and its nerves, but principally from the imperfect circulation which it must create in the spermatic vascular system. We

* Memorial des Hopitaux du Midi. No. 24.

need scarcely allude to the diagnostic marks of the disease, but we may be permitted to mention its more frequent occurrence on the left side, its subsidence under steady pressure, whilst the patient is in the recumbent posture, and the return of the tumor when the patient rises, although sufficient pressure is made on the abdominal ring to prevent the egress of a hernia.

With respect to the atrophy of the testicle, M. Delpech remarks that it frequently exists to some extent, when the variocele is not severe, and when careful examination is necessary to detect it. On reducing the variocele in such cases by steady pressure, the testicle itself seems to disappear also, leaving little but its membranes and vessels be hind, a circumstance that, on superficial examination, would hardly be discovered. What is remarkable is this, that when the disease had been relieved by the obliteration of the varicose veins, the size of the testis became gradually restored, and with the size the functions. The pain felt in cases of varicocele is not in the testicle itself, but in the nerves of the spermatic cord. M. Delpech relates five cases in order to display his method of treatment, and to elucidate the history of the malady. We shall briefly mention some of them.

Case. 1.—*Varicocele—Operation—Purulent Deposites in the Liver.*—A young officer of infantry was affected with variocele of the left side, and suffered so much pain and inconvenience, that he expected to be under the necessity of abandoning his profession. He had formerly suffered from intermittent fever, his complexion was sallow, his form spare. Under these circumstances he applied to M. Delpech, who performed the operation. The incision was smaller than in the former case, and the piece of amadou larger and stronger, in order that the constriction of the ligature might be slighter. On the second day the ligature was cut, and removed with the amadou. The veins formed a full and solid cord, and coagulation had taken place in them below the ligature; the lumbar pains had ceased. On the 12th day the wasted testis had regained some size, and there were erections of the penis; on the 21st the wound was cicatrized, the cord diminished in volume, and the testicle increased.

On the 24th day the patient was attacked with a rigor and attach of fever; his skin had assumed a slightly yellow tint. On the 25th and 26th days there were two attacks of fever, with rigors, thirst, and depression, but without any local pain or symptom. Quina was prescribed on the 26th, but during that day and night there were three attacks of fever, and in the evening of the 28th the unfortunate patient died.

*Dissection.* The cicatrix of the wound in the groin was firm, the cavity of the spermatic veins was obliterated, and they were solid and filled with a firm adherent clot. There was no plebitis nor inflammation of the peritoneum. The testicle was in a great degree restored, redder than natural, slightly "infiltrated," but otherwise healthy in structure. The liver was enlarged with prominences here and there, Uunder each of these prominences was one or several *foyers* of scrofulous tubercles of different degrees of consistence, for the most part in a state of softening. A great many abscesses existed deeply in the substance of the liver, and in their neighborhood the organ was inflamed.

Case 2. A cook, æt 26, had labored under varicocele on the right side for eight years. The pain was very considerable, and M. Delpech operated upon him. An incision, an inch in length, was made about an inch below the inguinal canal, in the direction of the cord—the varicose veins were exposed, seized with the forceps, and separated by the nails from the surrounding parts. A strip of amadou half an inch in breadth, was then passed under the vessels, and its extremities brought out at the wound, and secured there by adhesive plaster. Thus there was no ligature upon the veins which were merely isolated for a small space from the surrounding parts, and left in contact with the amadou. On the fourth day the veins were red externally, manifestly choked by the adhesive inflammation internally; there had been little swelling or inflammation of the parts around. The amadou was withdrawn. The success of this operation was complete, the cavities of the veins becoming obliterated, the disease cured, and the testis regaining its natural dimensions.

M. Delpech thinks the employment of the amadou a great improvement in the method of procuring obliteration of the veins. He thinks it cannot be attended with risk of phlebitis, although section and ligature of the vein certainly are so. We saw fatal inflammation of the saphena vein brought on in an adult, by rough examination only, though it is proper to mention that the patient had formerly had an attack of inflammation of the same vessel. Phlebitis also occurs spontaneously; therefore, we see no reason why M. Delpech's operation must necessarily be unattended with danger. In this country surgeons shew an indisposition to meddle with varicocele, and we certainly do not meet with many cases in which the inconveniences are very serious. We have laid the gist of M. Delpech's memoir before our surgical brethren, in order that they may put his proposal to the test of their experience, if fit and proper cases should present themselves. By the way, we may observe, that in one of the Parisian hospitals, the spermatic artery has been tied for varicocele, in order to effect the wasting of the testis. We should not expect much from this operation. Castration has also been performed, but on this we need say nothing. We again recommend M. Delpech's proposals and the result of his cases to the attention of our brethren.—*Medico-Chirurg. Rev.*

3. *On Death from Lithotomy.*—I cannot too strongly recommend to you the study of the cases that prove fatal. It is from them that you may learn how to prevent other cases from proving so.

*Too free division of the prostate* is one cause of death. What are the consequences of this too free division? At first the symptoms are not well marked. There is some heat of skin, quickness of pulse, want of sleep; but the more characteristic symptoms seldom begin later than 24 hours after the operation. They commence with shivering, pain in the loins—the pulse rises—the tongue grows white—the patient still retains his intellects. Now there is some pain in the pubes and left groin—the pulse has a weak beat and intermits, at first, perhaps, once in forty strokes, then in five or six—there is hiccup, and, if there was no shivering at first, there is shivering now. The belly becomes tympanitic, and in consequence of the tympanitis, tender. The symptoms go on—generally there is delirium, but occasionally the patient retains his senses to the last, and now death closes the scene.

*On dissection,* you find the intestines distended with air, but no general peritonitis, except just at the lower part of the belly, where the peritonæum is reflected from the rectum to the bladder, and where, perhaps, it is a little inflamed. The cellular membrane of the pelvis is sloughy, inflamed, putrid, and here is the mischief. You may think that peritonitis is common after lithotomy, and the profession generally do think so—but it is not the case. It is this *sloughing of the cellular membrane* that is frequent, and that is constantly mistaken for peritonitis. Don't make this mistake; if you do, if you bleed or purge, the clock runs down the faster. Give, at first, salines, with a little excess of ammonia; then opium, wine, brandy, &c. Adults very seldom recover—children occasionally do so. After presenting these symptoms for two or three weeks, one child recovered, a putrid abscess bursting into the wound. Another child recovered, a slough giving way into the rectum.

*Surgical Treatment of this Affection.* I will mention to you a case.

Case. In a patient whom I lithotomized, I used Mr. Blizard's knife, an abominable instrument. I cut through the prostate gland, and all seemed right to the lookers-on. I felt uneasy. In 36 hours there were suspicious symptoms; in 48 hours they were well marked: there were intermitting pulse, tympanitic belly, manifestly mischief in the cellular membrane, round the neck of the bladder. I introduced my finger into the rectum, and passed Savigny's probe-pointed bistoury up the wound to its left extremity; then I pushed forward the moveable sharp point, divided the coats of the rectum on my finger, and cut outwards with the point resting on my finger, exactly as you operate for fistula ani. The intermissions of the pulse grew less frequent, then ceased, and ultimately the patient recovered.

*Hæmorrhage.* This is another cause of death after lithotomy. I have myself seen three patients die of hæmorrhage. One was a patient of Sir E. Home's; he died when I was house-surgeon. A patient of an eminent lithotomist died. The third was a patient of my own, and in him the hæmorrhage was venous. I have heard of other cases, though I have seen no more.

*Division of Pudic Artery.* Many years ago, Sir E. Home operated on a patient for stone. In half an hour after the operation I found the patient apparently dying from profuse hæmorrhage, which was still going on. I brought the patient with his perinæum to the light, and finding that pressure on the pudic artery, stopped the bleeding, I tied it, by passing a curved silver needle between it and the bone. The bleeding was arrested and did not return. In a private patient the hæmorrhage was only stopped by an assistant's keeping his finger on the pudic artery for five or six hours together.

Sometimes there is hæmorrhage from the neck of the bladder. The best way under such circumstances, is to introduce the longest finger into the rectum, and press on the neck of the bladder. You should previously pass a canula into the wound; it serves for a point d'appui, and lets out the blood, &c.

*Death from Exhaustion.*—Case.—A patient underwent a tedious operation, the stone breaking, the perinæum deep, the man very fat. He was put to bed, and died with symptoms of exhaustion.

A long operation is always hazardous. Sometimes the patients fall afterwards into a deep dose or state of coma, and though they sometimes recover, they often die.

Persons affected with organic disease are, of course, not such good subjects for lithotomy, as those who are not so affected. But organic disease of other organs is not near so important as the sound or unsound state of the urinary organs.

*Enlarged Prostate.*—I do not think that an enlarged prostate is of any material consequence, for patients who have it do very well. But when the prostate is ulcerated, *never* operate. I have seen it done in three instances, and in each fatally.

*Chronic Inflammation of the Bladder.* Chronic inflammation of the mucous membrane of the bladder is a serious, but not a fatal objection. If the inflammation of the bladder is acute, they die.

Case. 1. Such a patient, on whom I operated, did pretty well for a week, then fell into a low state and died. The inflammation extended up the ureter, and muco-purulent matter was found in the pelvis and infundibula. I operated against my own judgment.

Case 2. Another patient died four or five weeks after the operation. There was inflammation and suppuration (?) in the cellular membrane *all* round the bladder.

*With Abscess in the Kidney.* If there be abscess in the kidney, the patient is sure to die. Inflammation of the kidney, independent of abscess, confers danger when there is pain in the loins and across the lower part of the belly—languor and utter listlessness—hands and feet chilly—urine albuminous; when there are these symptoms, the case is a bad one for operation. But if the urine contains pus or any thing approaching to it, the case is still worse. More frequently patients operated on with abscess of the kidney, die after a certain length of time, having apparently recovered from the operation.

Case. Sir E. Home operated on such a patient. He said during the operation that something had burst in the loins. He died in three days. On dissection an abscess *was* found to have burst into the cellular membrane, behind the peritoneum and below the kidney.

Generally they die several weeks afterwards with the usual symptoms of suppuration in the kidney.

*Age.* Children generally do well. Persons between 70 and 80 do better than those between 50 and 70. Such is my experience, and such is that of the Norwich Hospital.—*Brodie on Calculous Disorders.*

4. *Poisoning by Cantharides.*—A young woman serving in a dram shop, administered some lytta in raspberry brandy, to two young men, each of them taking half a pint of the liquor in question, One of them soon afterwards set out for Stourport, six miles distant, but was seized on the road with violent pain in the stomach and bowels, which compelled him to sit down frequently, insomuch that he was six hours in reaching the place in question. Mr. Williams, of Bewdley was summoned, and found him suffering from violent pain in the stomach, bowels, kidneys, and bladder; a constant desire to make water, and a burning heat in the throat. Vomiting was produced by a mixture of sugar, water, and sweet oil, and afterwards he was ordered to drink freely of linseed tea.

At 4. a. m. next morning, the skin was very hot, the sense of burning in the throat more urgent, the straining to make water incessant, whilst only a few drops of blood were voided, the pulse 100, the tongue thickly coated. *V. S. ad.* oz.xij.—*Enema,*—*ol. ric.* oz.iss. At noon the strangury continued, with much tenderness in the region of the kidneys, ureters, and bladder; bowels opened, pulse 100. *V. S. ad.* oz.xiv.—*Rep. ol. ric. et enemata. Linseed tea and barley water.* At 5, p. m. the tenderness was much increased; pulse 115; great restlessness, with occasional delirium. *V. S. ad.* oz.xvj.—*Ol. ric. et enemata.* 12, p. M. Restlessness very great; constant delirium with some slight convulsion; face much flushed and conjunctivæ injected; strangury distressing; pulse 125, hard and incompressible. He was bled to oz.xxiv., kept in syncope for nearly an hour, then allowed to lie down, and eight drops of laudanum in some linseed tea were given him. After this the urgent symptons ceased, though the urine continued somewhat bloody, and the tongue peculiarly coated till the 6th or 7th of July. The patient was then convalescent.

We need not give the particulars of the other case, as the symptoms were similar to those of the last, and the same kind of treatment, though not so active, proved successful.—*Medico Chirurg. Rev.*

5. *Congenital Malformation of the Cornea and Sclerotica.*—A servant, æt. 22, has a small excrescence on the left eye, arising from the cornea and sclerotica, at its temporal side; its surface white, smooth, convex; in its centre a small depression from which strong black hairs arise, which incline downwards, so as to hang over the lower lid; it is firm to the touch, and appears to be covered by the sclerotic conjunctiva; the surrounding cornea and sclerotica preserve their natural structure. He says that the blemish was congenital, and that the hairs began to plague him only a few years ago. The man usually calls on Mr. Middlemore once in every six weeks, in order to have the hairs removed. Mr. M. intends to apply the nitrate of silver to the cavities containing the bulbs of the largest hairs, when he next extracts them, and if this fails to dissect them out, if it can be done without danger of penetrating the globe. The hairs are of the same color, and have the same inclination as those of the lower eyelid. This is the only instance of such a congenital malformation which Mr. Middlemore has witnessed.

In a patient with hernia we remarked a deficiency of the circle of the iris at its inferior part. The pupil was of course oblong from above downwards; vision was very imperfect with that eye. The same individual had all the appearances of the blue disease, though not in an aggravated degree, so that there seemed to be a tendency to imperfection in her formation, a circumstance not uncommon.—*Mid. Report.*

6. *Treatment of Croup.* Speaking of the remedies for this disease, Mr. Goselad remarks, "Amongst these, the warm bath is one of the most active, and, at the same time, most injurious; and I cannot imagine how any one, who has once witnessed its effects, can again recommend it in Croup. It is, in my opinion, so decidedly hurtful, by quickening the circulation, that I should interdict its use in almost all inflammatory cases. The warm bath, I think, is never useful unless prolonged until faintness is produced; and in the early stages of inflammatory complaints, it is often impossible to produce this effect, until the heart beats more than 130 times in a minute, which is a degree of excitement I think unwarrantable. If resorted to later, effusion is brought on sooner than it would otherwise supervene; and many practitioners could, I think, call to mind cases, where its use has been followed by unexpected death: the vessels previously emptied perhaps by bleeding, having given way, and appoplexy supervened." —*Med. Chirurg. Rev.*

7. *Vesico-Vaginal Fistula successfully treated by the Suture.* Marie Reggiani, æt. 22, had labored under a vesico-vaginal fistula since her first confinement, which had been protracted and severe; the finger could be readily passed through the fistula into the bladder. For eight months a great variety of plans were employed without success, and she repaired to M. Malagodi at Bologna, who performed the operation we are about to describe. Having placed the patient in the lithotomy position, he introduced the finger of the right hand, covered with the finger of a glove, into the fistulous opening, and bending it drew down the left border of the fistula to the orifice of the vagina; and then, with the other hand he pared off the border with a straight bistoury. The hand was changed, and the same operation repeated on the right border of the fistula. The object was now to bring the two pared edges in contact. M. Malagodi had armed each end of these ligatures with a very small curved needle, and there was a handle to which the needles might be fixed at pleasure. He then reintroduced the right index finger into the opening, and brought down its left border, and with the left hand passed a needle through it near the posterior angle of the wound. A second and third needle were passed in the same manner, at equal distances from each other, and their opposite border was transfixed in the same manner, when the ligatures were tied, and the sides of the opening brought together. The patient was placed on her back in bed, and the catheter kept in the bladder, and the urine allowed to flow constantly into a porringer. Until the morning of the third day the water was passed through the catheter only, but then it began to escape by the wound into the vagina. On the fourth day an examination was made by M. Malagodi, who found that the two posterior sutures remained firm, and on withdrawing them the union of the sides of the fistula was complete. The anterior ligature on the other hand, had torn the left border of the wound, and nearly a third of the original fistula thus remained unhealed. To effect the contraction and perfect cicatrization of this, M. Malagodi touched the edges regularly with the nitrate of silver, leaving the catheter constantly in the bladder. The plan was persisted in for some weeks, and was crowned with perfect success.—*Rocoglitor Medico, July,* 1829.

8. *Mr. Duffin's New Shield Pessary.* Mr. Duffin has invented a pessary, which he thinks superior to those in common use. It is a modification of the stalk pessary, and by means of a graduating screw, the length of the stalk can be accommodated to that of the vagina as the cure advances. The head, which is hollowed at its summit into a cup, being of a size adapted to that of the vagina in the particular case, and hollowed in proportion to the magnitude of the parts it has to receive, affords support without irritation or injury. The head may be made to unscrew, so that as the vagina contracts, a smaller one may be substituted for it. The shield being pressed externally against the perinæum and perinæal part of the labia, and retained firmly in that situation by a thick T bandage of cotton, to which it may be attached, supports steadily through the medium of the head and stalk the weight of the prolapsed organ, and obviates all vacillation of the extremity of the stalk. Between the shield and the stalk is a ball and socket-joint, allowing the instrument to accommodate itself to every change of posture, preventing any jarring impulse or undue friction. The pessary being hollow, and open at the extremity of the stalk, the shield also being perforated in this situation, whilst the head of the instrument also being perforated in numerous places, any astringent or other lotion can be thrown in by means of a syringe or India-rubber bottle. The instrument has received the approbation of Drs. Charles Clark, Bundell, Henry Davies, and Robert Lee. The pessary is best made of ivory, but for economy, the head and shield may be constructed of boxwood, the stem and joint of ivory. The instrument may be procured from Messrs. Stoddart, No. 401, Strand. A woodcut representing it is to be seen in the Medical Gazette for March 26th, 1831.—*Medico-Chirurg. Rev.*

9. *Congenital Blindness cured at an advanced Age.** When this lady came under Mr. Wardrop's care, she was in her 46th year. The right eye-ball was collapsed, the left retaining its natural globular form. The cornea of this eye was transparent, except at one point where there was a linear opacity, probably the cicatrix of a wound made by a Parisian oculist, in an unsuccessful attempt to restore vision when she was six months old. The anterior chamber of the eye was of its natural capacity, but no vestige of a pupil was perceptible, some streaks of yellow lymph being deposited in an irregular manner over the central part of the iris. There was reason to believe that the retina was sound; for though she could not perceive objects, or form any idea of colors, yet she was able to distinguish between a very light and a very dark room, and between a gloomy day and sunshine. Under this impression, Mr. W. thought the attempt to restore sight by an artificial pupil was worthy of a trial. On the 29th January, he introduced a small needle through the cornea, and passed it through the centre of the iris, but could not destroy the adhesions which had shut up the pupillar opening. After this she could perceive a little more light, but not forms or colors. On the 8th February, a second operation was performed, by passing a sharp-edged needle through the sclerotica, bringing its point through the iris into the anterior chamber, repassing it into the posterior chamber at a small distance, and then dividing the portion of iris thus included between the two perforations of the needle. A slight inflammation followed—the light became offensive—but it was evident the sight was still very imperfect. On the 17th of February, a third operation was performed, which consisted in still further enlarging the opening in the iris, and in removing some opake matter by a needle introduced through the sclerotica. In returning home from Mr. Wardrop's house, with the eye covered with a loose piece of silk, the first thing she noticed was a hackney coach passing, when she exclaimed,—"What is that large thing that has passed by us?" In the course of the evening, she requested her brother to shew her his watch, which she narrowly surveyed close to her eye. She remarked there was a dark and a bright side. She pointed to the hour of 12 and smiled. When asked if she saw any thing more on the dial plate, she replied, "yes," and pointed to the hour of 6, and to the hands of the watch. She then looked at the chain and seals, remarking that one of the seals was bright, which was the case, being of crystal. Next day she refused to look at the

* Case of a lady born blind, who received sight at an advanced age by the formation of an artificial pupil. By James Wardrop, Esq. F. R. S. Ed. &c.—from the Philosophical Transactions.

watch, observing that she was confused by the visible world thus for the first time opened to her. On the third day, she observed the doors on the opposite side of the street, but mistook their color. This day she saw the nose on her brother's face, (dimensions and color not stated,) and on the sixth day saw still better, but was bewildered, from not being able to combine the sense of sight and touch, feeling disappointed in not having the power of distinguishing at once by the eye those objects which she could easily distinguish from one another by feeling them. In short, she had almost every thing to learn—for although she could distinguish one object from another, she knew not, by the newly acquired sense, what any object was—nor could she form any idea of its distance from her. She was, however, highly delighted with restored vision, and we almost envy her the pleasure which she must feel on the sight of so many *novelties*, at an age when novelties begin to be scarce articles with most human beings—even of her sex.

There is no other case on record where the sense of vision was restored at so late a period of life—and it is not a little remarkable, that the optic nerve should remain fit to receive the impressions of external objects for the long space of 46 years, during which, it was totally excluded from the performance of its function. The operation is very creditable to Mr. Wardrop, and we wish him long life and many opportunities of *enlightening*, not only the visual orbs of his fellow creatures, but the dark paths of the medical science. We also wish him plenty of "*pupils*," natural and artificial, to operate on in Bartholomew Close—which some folks think is rather *too close to* Bartholomew.—*Ibid.*

10. *Œsophagitis Hydrophobia.** The following case proves one of two things—either that the bite of a dog (which was not proved to be mad) could be followed two years afterwards by symptoms of hydrophobia; or, what is much more likely, that hydrophobia may result from other causes than the bite of a rabid animal.

Case. J. Kneiss, aged 48 years, of sanguineous temperament, and in excellent health, received a slight scratch from a dog, that did not evince, before or after, any appearance of hydrophobia. He took no notice of the accident. Nearly two years after this, having drank freely of cold liquids, while heated by work, in the month of May, he soon experienced a difficulty of swallowing fluids, a sense of weight in his limbs, and a feeling of intense cold throughout his body. He took some trifling medicines, and on the third day there was violent fever great thirst, and such a spasmodic affection of all the parts about the œsophagus and larynx, on the attempt to swallow liquids, that he fell into a kind of deliquium. This was reproduced by each succeeding attempt. On being received into the hospital at Bamberg, he presented, besides the foregoing symptoms, a remarkable velocity in all his move-

* Ch. Pfeuser, Senior Physician to the General Hospital of Bamberg.—*Heid. Klin. Annas.* 1825.

ments, a loquacity of speech, and a furious expression in his countenance. The pulse was quick and full, the action of the heart precipitous, and sometimes suspended, skin hot and dry, thirst considerable, tongue coated, urine scanty, red, and turbid; deglutition of solids easy. He was bled to thirty-two ounces, and had a laxative enema. The ensuing night was spent in dreadful agitation, with occasional delirium, convulsive movements, and fits of lipothymia. The pyrexia now ceased for a time, and calomel with belladonna, in small doses, were prescribed, while mercurial frictions with the same were applied along the spinal column. The thirst soon afterwards became intense, the pupils dilated, and a frothy mucus was ejected from the mouth. These symptoms continued for two days more, and the sight of water or any other liquid produced horrible convulsions. The patient complained of intense pain in the region of the stomach, and the tongue seemed inflamed. Eight ounces of blood from the jugular vein, and twenty leeches applied to the head. Ice was also applied to this part, a blister to the nape of the neck, and sinapisms to the lower extremities. In the evening, a severe paroxysm of agitation, and 32 ounces of blood were abstracted. A complete remission of all the symptoms followed, the patient was calm and collected, full of hope, and swallowed whatever medicines were offered to him. This state of tranquillity lasted all the day, and in the beginning of the night he had three hours' calm sleep. From this period he fell into a state of extreme apathy, stupor, and complete prostration of physical and intellectual forces. He died in a few hours afterwards.

*Dissection.* The cerebral substance was extremely soft—spinal marrow sound—salivary glands very much swelled—some gangrenous eschars on the surface of the pharynx and larynx, the vessels of which were extremely injected—thyroid gland enlarged, changed in color, soft, and full of thin black blood—œsophagus inflamed throughout its whole extent, and presenting some spots of gangrene—the diaphragm in the same condition—mucous membrane of the stomach and duodenum very red—pancreas in the same state as the salivary glands.

This is a curious case; and we think, there can be little doubt that the ingurgitation of cold liquids, when the body was heated, produced the inflammation in the mucous membrane of the parts above-mentioned; and that this inflammation, tending so rapidly to gangrene, gave evolution to the whole of the phenomena described. The more we study the physiology and pathology of these mucous membranes, the more we shall be astonished at the overwhelming influence which irritation of their nerves exerts over the whole of our physical and intellectual functions. This study is yet in its infancy, and it presents the widest, as well as the richest field for genius and talent to cultivate—*Ib.*

11. *Case of Extra-uterine Fœtation.* By M. F. Wagstaff, Esq.;—"On the 28th of April, 1829, I was desired to visit Mrs. Rondeau, a married lady, about 25 years of age. I had delivered her of twins in her first accouchement, and of a single child in her second, which child, at this period, had not been weaned. No irregular circumstances occurred in either of those labors.

I found her reclined on a sofa, on the right side, her countenance very pallid, pulse greatly accelerated and small, much anxiety about the præcordia, lancinating pains in the abdomen, constant tenesmus both of rectum and bladder, with frequent vomiting.

Her mother gave the following statement of the previous occurrences:—

"After having eaten some cold lamb and potatoes, and drank a glass of porter, we walked together into Blackfriars' road, (about half a mile,) when Mrs. Rondeau suddenly complained of acute pain in the body, and requested a coach to be procured, in which she was conveyed home, where I gave my daughter some warm brandy and water immediately, supposing the pain to have arisen from indigestion, especially as it was attended with vomiting and relaxation of the bowels, and applied warm fomentation: this took place at two o'clock; at four, (as I found the pain increase,) I sent for you."

The symptoms, as they appeared on my arrival, I have just described: they were very analogous to those of Cholera Morbus; but as no inflammatory symptoms supervened, I prescribed anodyne and carminative medicines.

The pain continued during the whole evening; the abdomen enlarged, the pulse diminished, and, a little before twelve the same night, she expired.

The rapid progress and fatal termination of this case naturally excited a desire to be better acquainted with the *cause* of dissolution, which was not clearly shown by the symptoms; I therefore requested permission to inspect the body, which was readily granted by the relatives.

On the subsequent afternoon, I went accompanied by Mr. Nordblad, Curator of St. Thomas's museum, and my son, to make an examination, of which the following is a statement:—

*Sectio Cadaveris.* On the appearance of the body, fourteen hours after death, the surface exhibited a completely blanched state, with fulness and tension of the abdomen.

On cutting through the abdominal muscles, the peritoneal sack was found to be distended with *blood*, (amounting, by weight, to four pounds fourteen ounces,) partially coagulated.

While this was being removed cautiously, a fœtus was discovered in it of about seven or eight weeks' growth.

No laceration appearing in the uterus, attention was naturally directed to the appendages of that viscus, when the part where the fœtus had escaped was manifested by the ragged placenta, which hung from its situation in the left fallopian tube.

This left no doubt as to the nature of the case. The parts were then removed, and on a careful examination, it was ascertained that the fœtus had been contained in a sac formed by the left fallopian tube.

This sac was equal in size to a pigeon's egg, and occupied two-thirds of the length of the tube, nearest to the uterus.

The remaining portion of the tube was of its natural size, and its fimbriated margin free from obstruction.

A probe passed along its canal, stopped abruptly at the commencement of the dilatation, where the placenta protrudes; the distention of its sac increased to its centre, at which part it had given way, allowing the exit of the fœtus.

The rupture is as large as the point of a finger, and through it a detached portion of the placenta hung into the abdominal cavity.

The body of the uterus had enlarged to a size equal to that of the same viscus at a similar period of gestation; its walls were thickened and its cavity lined by recent deposit, evidently forming the decidua.*

A careful examination could not detect any rent in the peritoneal surface, nor was there the slightest mark of visceral disease.

*Remarks.* Extra-Uterine Fœtations are probably more frequent than we have been accustomed to imagine: little was known about them previous to the last century.

Mr. Turnbull, a surgeon of eminence, published a case of Extra-Uterine Fœtation, of the ventral kind, where pregnancy had existed fifteen months, and the child was (*post mortem*) removed by him from the abdomen. In this publication he refers as far back as 1683, to a case published in the Philosophical Transactions of that date, and gives a list of references to various authors, down to the days of Drs. Smellie, Lowther, and Garthshore, and so on to his own case, published in 1791.

He remarks, 'that although it is generally understood that the uterus is essentially necessary for the purposes of conception, yet these different fœtations incline us to believe that it is not *absolutely so*, and that the principal advantages which that organ possesses over other living parts are derived from its situation and dilatable powers, and from its being possessed of *muscular* structure wi‘h an *external opening*. The former being admirably calculated for the purposes of growth and evolution, without any interference with the vital parts: and the latter for the prevention of hæmorrhages and the expulsion of the fœtus.'

The case I have just related seems calculated to corroborate this hypothesis. Does it not prove that the uterus is only a receptacle? though evidently the best suited for the purpose (being the natural one); yet, in this particular instance, the fœtus was nourished seven or eight weeks in its unnatural habitation—the fallopian tube, which, being impervious at the uterine extremity, could not permit the passage of the fœtus into the uterus.

* "This is a peculiar circumstance, worthy of investigation. How does it happen that the decidua should be *forming in* the uterus, when the fallopian tube was imperforate *beyond* the fœtus? No communication whatever between it and the uterus. The uterus had taken on its action of pregnancy, although empty, and a deposit to form, or forming a membrane.

A question here naturally arises, How was the fœtus nourished? It continued to enlarge until the texture of the fallopian tube gave way to over-distension.

It is evident that its nourishment was obtained from the vessels of the placenta, which received it from their numerous ramifications on the internal surface of the fallopian tube. Suppose, in the expulsion of the fœtus through the rupture in the fallopian tube, the funis umbilicalis had *not* been separated, but remained perfect, would it not have continued to supply the fœtus from the same source it had done before, namely, the internal surface of the fallopian tube; and might not its ramifications have been extended over neighboring parts, and thereby produced a supply augmenting with the enlargement and demand of the fœtus? The generally received theory is, that the ovum is impregnated in the ovarium and becomes detached in consequence; the fimbræ of the fallopian tube, having seized the ovarium, convey the ovum, at the moment of its detachment into the uterus, for nourishment; but, if they should miss their grasp,—or, having obtained it, should lose their hold, it must fall into the abdominal cavity, and thereby constitute a ventral case.

Adhesion readily takes place, from which ramifications are soon found to extend, and obtain a supply for the nourishment and growth of the fœtus.—*Medico-Chirurg. Rev.*

12. *On Death from Syncope—Observations on Venesection.* By M. Piorry.—The cessation of the cerebral circulation is, our author thinks, next to asphyxia, from the accumulation of bronchial mucus, the most common way in which death takes place. In all great hæmorrhages there is an abundant secretion of fluid from the surface of the intestinal canal, and also from all the other mucous tissues, which tends further to exhaust the strength and deprive the brain of a due afflux of blood. The heart being ill-supplied with nervous energy from the brain, becomes distended, and at length ceases to carry on the circulation. From the circumstance that the brain is encased in bone, the vessels will be found full after death, even from hæmorrhage—and this has led some pathologists astray. Our author has often seen death occasioned by injudicious venesection. In dilatation of the heart at the Hotel Dieu, he has often seen the fatal effects of blood-letting. The author therefore lays down a set of rules respecting venesection, which he thinks it would be of great use to observe.

1mo. When, in a person, we find a dull sound on percussion of the chest, when there is active dilatation of the heart—when the liver is enlarged—the veins distended, the pulse full, and the circulation active, we may be sure that the circulation is in excess, and we need not fear to bleed freely. But if the symptoms are the reverse of the above, we should be cautious in the use of the lancet. We should also be cautious, he observes, when the skin is fresh-colored, but where the internal organs give indications the reverse of plethora. In doubtful cases, we should never bleed in the horizontal position; for if syncope then occurs, we have no recourse from the change of posture.—*Ib.*

13. *Baron Heurteloup on Lithotrity.—Construction of Instruments.*—"First Rule. Rectitude in a sound being: 1st, *the most favorable shape for bringing, without effort, the extremity of the instrument into contact with a great extent of the parietes of the bladder, and more especially with its fundus;* 2d, *for allowing the instrument to be removed backwards and forwards with facility;* 3d, *for permitting it to be rotated on itself,* all instruments of lithotrity *must be straight in that portion of them which remains in the urethra during the operation,* for a curve would render them faulty in proportion as it impeded the execution of these three fundamental properties.

Second Rule. Since the operation of lithotrity must be performed whilst the bladder is distended with water, it follows that the instrument must unite the necessary conditions for preventing the liquid, when once introduced, from escaping during the operation; hence, 1st, *it is necessary that the instrument should be, as nearly as possible, of the same diameter throughout, and that its vesical extremity should be as little enlarged as possible,* for the urethra through which an end much enlarged passed, would be but incompletely filled by the body of the instrument, and the water would consequently escape between the instrument and the sides of the canal; 2d, *the instrument must be of sufficient size to fill the canal, but not large enough to distend it, and render the movements difficult;* 3d, *it must finally be constructed so as to prevent the water from flowing out between its component parts.*

Third Rule. Since the urethræ vary in size, from two lines and a half, to four and a half, it follows *that the instruments must also present different diameters.*

Fourth Rule.—The contraction of the bladder being sometimes powerful enough to expel, between the canal and the instrument, almost all the water it contains, it is necessary *that every instrument of lithrotrity should allow a fresh injection to be made into that organ, in order to distend it,* so that the instrument may be closed without any danger.*

Fifth Rule.—Since the bladder varies in its anterŏ-posterior diameter, from two to three inches, on account of its contraction, *a lithotritic instrument must be constructed, so that its branches may expand sufficiently to seize the stone, and yet, not project more than two inches and a half from the tube which contains them.* The instrument must also admit of *the projection of the branches being diminished if circumstances should require it;* and this is the more requisite, since those bladders which contain the largest stones are generally the least capacious.

* "I am here only alluding to the instruments which act by *progressive wasting* on the stone, and which consist of three or four branches, for these alone are apt to become entangled in the bladder."

Sixth Rule.—When an instrument is expanded, and is in action in the interior of the bladder, the spot where the branches project from the tube is almost always in close contact with the neck of the organ; hence it is necessary *that the brancher should have free motion, without affording a possibility of pinching at the part where they unite, and should always preserve the same distance between them, whether projected from, or drawn into the tube.*

Seventh Rule.—The bladder sometimes violently contracts during the operation of lithotrity, aud presses upon the branches of the instrument which, when drawn towards the neck, are compressed by this part of the viscus, so that the surgeon is no longer master of the expansion of his forceps, and his manœuvres are totally impeded. It follows, therefore, *that the branches of every liihotritic instrument should be supported when they project from the tube and are expanded in the bladder, and that their expansion should not be entirely entrusted to the elasticity of the metal.*

Eighth Rule.—The object in constructing a lithotritic instrument being to bring a mechanical agent into action in the interior of the bladder, where the surgeon is unable to see in what manner it acts; it follows, *that every instrument for lithotrity must be graduated;* so that by means of certain signs placed at the extra-vesical extremity of the instrument, *the operator may be enabled to ascertain what is going forward at the intra-vesical portion,* Marks well contrived, are therefore of the greatest importance.

Ninth Rule.—Some bladders are very irregular, and their superior and posterior parts, although distended with water, are sometimes forced from the sacrum towards the neck of the bladder, either by the contraction of the viscus itself, or by the pressure of the intestines which are pushed down by the abdominal muscles; it follows, therefore, that an instrument, destined to seize large calculi, which require a large expansion of the branches is faulty in its construction, *if the branch which is above, terminates in a hooked extremity; it is the more faulty the further the branches project from the tube to grasp the stone, and the smaller the size of the bladder.*

Tenth Rule.—Since it is necessary under certain circumstances, to discontinue the operation of lithotrity, either on account of a great degree of sensitiveness in the patient, or because of a violent and permanent contraction of the bladder, it follows, that it is not sufficient for an instrument to *grasp* the stone with facility, *but it must relax its hold with equal ease;* and as this becomes more frequently necessary in operating on large calculi, this property must predominate more especially in those instruments destined to act in such cases.

Eleventh Rule.—Since the excavation of large calculi produces a very considerable quantity of powder, which would clog the action of the 'evideur' (excavator), it follows, that *an instrument intended to excavate such calculi, must afford the facility of injecting water into the bladder to wash away the superabundant powder, even whilst the instrument is in action.*

Twelfth Rule.—Since that portion of the instrument which destroys the stone by *progressive wearing away* must be removed as far as possible from the parietes of the bladder, it follows *that this action must proceed from the centre of the calculus to its circumference;* for were it otherwise, the destroying agent would be in almost immediate contact with the interior of the organ.

Thirteenth Rule.—Since the action of the instruments upon the stone must be performed with the greatest regularity, both as regards the action of seizing and that of breaking, and since the exact performance of this must depend entirely upon the manœuvres of the surgeon, it follows, that *no part of a lithotritic instrument must be left to act by means of springs, the movements of which are in a great measure independent of the surgeon's will.*

Fourteenth Rule.—Since it is necessary that the different parts of an instrument should play with facility, one upon another, in order that the surgeon may have all the precision and delicacy of his tact, it follows that the instrument *must not only admit of this free and easy play*, but it must also be so constructed as to render it easy *to take it to pieces, so that it may, in all its parts, be thoroughly cleaned.* Without the observance of this condition in an instrument, there cannot be sufficient precision in the manœuvres."*

*On the Circumstances which interfere with, or are favorable to Lithotrity.*—Lithotrity is not equally applicable to all ages. It is generally, and for reasons which must partly be very obvious, inapplicable to children under eight or ten years of age. The adult is the most favorable time of life for the operation, and the smaller the stone, and the earlier it is operated on the better. Old persons are less adapted for lithotrity on account of the deficient power of the bladder to expel its contents, and the fragments of the calculus.

Corpulency, which is an impediment to lithotomy, rather offers facilities for the performance of lithotrity; it is therefore no objection, at least in a healthy condition of the urinary system. A great degree of organic disease furnishes sufficient reason for shunning the operation, especially if several sittings are required. A strong cal-

* "A great number of instruments have been invented to perform the operation of Lithotrity, and more especially in France, but they have proved unfit for use, as the most part of them deviate from one or the other of the rules here laid down, and are thus the more defective and inapplicable, in proportion to the importance of the rule neglected; many of these instruments are faulty from the non-observance of 5, 6, 7, or 8 rules, and unfortunately operations attempted with them have sometimes caused the death of the patient; but can such fatal terminations be laid to the account of Lithotrity?"

culous diathesis may also prohibit lithotrity, or give the surgeon pause before he ventures on it.

The state of the urethra requires consideration. If naturally of small size, the circumstance is unfavorable; a small orifice is inconvenient, and must be divided, and stricture of the urethra renders it difficult or even impossible.

A large scrotal hernia is an embarrasing, but not insurmountable obstacle.

"Stones lodged in the urethra give rise also to great, and sometimes even insurmountable difficulties, especially when the calculus has become imbedded in the canal, and can only be removed by an opening. * * * There is a very important consideration with regard to calculi thus lodged in the urethra, to which lithotomy itself gives rise. Before the art of pulverizing a stone in the bladder was introduced, the rule was, that, when a calculus became lodged in the urethra, great care was to be taken not to push it back again into the bladder, but to extract it by making an opening into the canal. This rule was given in order to avoid the operation of lithotomy, which must be performed if the stone be made to pass from the urethra into the bladder. But now that we are enabled by more gentle means, to remove a stone, thus returned into the bladder, without subjecting the patient to the dangers of lithotomy, the rule ought to be precisely the contrary—that is, the stone ought, if possible, to be returned into the bladder, and no opening made to remove it; for, if this operation be trivial when compared with lithotomy, it is an important one when compared w ith lithotrity. The advantages of lithotrity are still more importa nt when we consider, that, when a stone, thus lodged in the ure thrahas been removed by an opening, it is frequently found necessary to perform lithotomy besides, to remove other small calculi remaining in the bladder, the existence of which was not suspected, since the stone lodged in the urethra obstructed the passage, and prevented those in the bladder from being ascertained."

A soft and fungous state of the mucous membrane of the urethra is disadvantageous for lithotrity; a very sensitive urethra is so also; bnt this extreme sensibility may generally be relieved or removed by the daily introduction of flexible bougies.

The state of the prostate has great influence on the performance of lithotrity. When enlarged it impedes the operation, and its various varieties of form and figure exert a material influence on the operation. "Not to be unjust, however," says the Baron, "towards the prostate gland, I will here terminate by remarking that it is too often accused of producing difficulties which result from totally different causes."

The condition and form of the bladder are, of course, of great importance. A large lateral diameter of the bladder is productive of great inconvenience and difficulty. Its form is sometimes injuriously altered by internal hæmorrhoids, or accumulations of fæces in the rectum. A bladder presenting pouches requires great care in the manœuvering of the instrument, and when it contains two or three cavities communicating with each other, it offers great difficulties both to the lithotritist and the lithotomist. A bladder of moderate size is the best adapted for the operation. A diseased state of the bladder deserves great attention. Chronic inflammation of the mucous membrane is common, and gives rise to considerations of importance.

"The first of these is, that a patient seldom has a stone and a catarrh of the bladder, without the latter being symptomatic of the former; it is only when the stone has existed for a long time, when the bladder is contracted and thickened, that the catarrh becomes idiopathic, that is to say, that it does not cease when the stone is removed. A proof of this is found in patients who have undergone the operation of lithotomy, and have been cured, but still retain a catarrh of the bladder after the wound has healed. These examples are rare, because, when the bladder is thus catarrhal, hypertrophied, and much diseased in its structure, it is seldom that patients recover after lithotomy. As long as the diseased state of the mucous membrane is not combined with a diseased state of the muscular and cellular tissues, producing the thickening of the coats of the bladder, and that rigidity which prevents its being distended, catarrh does not prohibit the operation."

When the urine is muco-purulent, with a disposition to hæmorrhage, the operation is prohibited.

"Thus the mucus of the bladder only prohibits lithotrity, when the stone is very large, and when there is also contraction of the organ, arising from the thickening of its coats; it is quite clear, that if the stone be small, though the bladder be catarrhal and contracted, lithotrity is practicable, not only on account of the absolute smallness of the stone, but because, being small, it can only be of recent formation, and the contraction of the bladder is not caused by the thickening of its coats, but by the irritation arising from the presence of the calculus, which is then most commonly light, friable, and easy pulverized, but presents small crystals which resemble needle-points. When speaking of the different kinds of calculi, we explained the reason why stones of this sort caused so much irritation and catarrh of the bladder in so short a time."

Paralysis of the bladder offering an obstacle to the discharge of fragments is very inconvenient. Vesical growths and polypi are occasionally met with, and require great care and prudence on the surgeon's part. Sometimes the mucous membrane of the bladder is in a soft fungous state, one very disadvantageous to lithotrity. A varicose state of the veins at the neck of the bladder is unfavorable, and various growths or diseases around it are so likewise. In some patients the bladder is extremely contractile; this is obviously more or less inconvenient. Finally, the Baron winds up with a sort of summary of what the lithotritist should be, and what he may expect.—*Med. Chirurg. Rev.*

14. *Remarks on Lactation; with Observations on the Healthy and Diseased Conditions of the Breast-milk, &c., &c.* By Edward Morton, M. D., Cantab. Octavo, October, 1831.—Some part of this work was published four years ago in a contemporary journal, and we are informed by the author that subsequent experience in the infantile diseases has convinced him of the correctness of the views which he then broached. The volume is divided into three chapters, the first of which contains nothing that need detain us here. The second is on the disorders that are frequently produced by lactation.

"In cases of extreme delicacy of constitution, lactation will often produce the worst effects. Many young ladies, on becoming mothers, are incapable of supporting the constant drain to which the wants of their infants subject them—they lose their good looks, become gradually weaker, and as their strength declines, their milk is simultaneously lessened in quantity, and altered in its other properties.

If the suckling be still continued, their debility daily increases, distressing pains in the back and loins succeed; the patients become exceedingly nervous, as it is termed, and are unusually susceptible of ordinary impressions; pain in the head, often of great violence, follows, which, in same cases, is succeeded by delirium, in others, by absolute mania. Nor is this the whole catalogue of ills to which, in such cases, the unfortunate mother was subjected: the appetite fails, distressing languor is experienced by day, while copious perspiration, which may easily be mistaken for, or terminate in, true pulmonary consumption; finally, the sight becomes progressively weaker, until vision is almost destroyed; the eyelids exude a glutinous secretion, and ophthalmia itself is occasionally induced.

These are the symptoms too often caused by lactation in delicate or debilitated habits, even a few months after delivery; the same also are observed when suckling has been injudiciously protracted beyond the period to which it should be confined.

A few only of the foregoing symptoms may be noticed, or nearly the whole may present themselves, in the same patient, and when this happens, unless the cause which has given rise to them be at once detected, and appropriate treatment applied, the most serious consequences may be apprehended."

In such cases, Dr. M. observes, half measures are useless or worse. Suckling must be discontinued altogether, for reasons which the author states, and which are sufficiently cogent. The obvious dependency of the foregoing symptoms on debility, will lead the practitioner to the employment of tonics, and other means of invigorating the constitution. Injudicious practitioners have sometimes recourse to cupping for the headach, which, of course aggravates the evil. The occurrence of miscarriage from suckling is illustrated by a case in private practice; and the author says that it is made public at the desire of the patient herself. We shall quote this case.

"Mrs. A———, a lady of delicate constitution, about twenty years of age, three or four months subsequent to the birth of her first child, began to find her milk gradually lessen in quantity; it had also much changed from its previous appearance, resembling at the time just stated, a yellowish, turbid serum, her child became emaciated; and diarrhœa supervening, my professional services were required. My advice was, that the child should be at once weaned, and a suitable wet nurse, if possible, procured—neither of which suggestions, as will shortly appear, were followed. I urged the necessity of this measure more particularly, because Mrs. A. was daily getting thinner and weaker; she also complained of great pain in the head and back, and of an increasing dimness of sight, which made her fear she should become blind; but the mother-in-law of my patient being, unfortunately, of opinion that pregnancy in the latter would not again occur during the continuance of lactation, recommended that the child, although chiefly supported upon spoon-meat, should occasionally be allowed to take the breast,; and this plan, notwithstanding the wish of Mrs. A——— to the contrary, and my own remonstrances on the subject, was adopted—the effects of which were to increase the mother's ailments, as well as those of her infant. Things went on thus for some time longer, when I once more endeavored to persuade Mrs. A——— to follow my advice, observing, that by an opposite line of conduct she was not only injuring her own health, but that of her child, neither of which I assured her, in my opinion, would be re-established till the latter had been weaned. I expressed also, my complete incredulity as to the non-recurrence of pregnancy in consequence of her infant remaining at the breast; and I added—'It is my firm conviction that if you be pregnant, or should happen shortly to become so, you will miscarry.' About a week after the conversation, she was suddenly seized with flooding, and what I had predicted took place. She now left off suckling, and in about a month, under suitable treatment, completely got rid of all her former complaints; the child also immediately began to improve."

The third chapter embraces the subject of disease in children, resulting from protracted lactation. To this source our author traces cases of vomiting, diarrhœa, debility, scrofula, tabes, convulsions, epilepsy—and, lastly meningitis, with hydrencephalus. This is a long and frightful list of maladies sucked from the breasts of our mothers! It is to meningitis, however, that Dr. Morton chiefly directs his attention. The conclusions to which our author has come, from experience, are as follow:—

"1st.—That if children be suckled for an undue length of time, they will be liable in consequence to be affected with meningitis, or inflammation of the investing membrane of the brain.

2dly,—That should they not become affected with the disorder in question during or soon after the time they are thus improperly suckled, they will nevertheless acquire therefrom a predisposition to cephalic disease at some further period of their lives.

3dly,—That children who are suckled for an undue length of time, when laboring under other diseases, will be much more liable to have the head secondarily affected, than children brought up in a different manner.

4thly,—And lastly, that the same effects will take place in infants, if suckled by women who have been delivered an undue length of time; although the infants themselves may not have been at the breast for too long a period."

15. *Reunion of Divided Parts.*—Baron Larrey, in his highly interesting volume of Surgical Memoirs, of which a translation has recently been published by Messrs. Carey and Lea, relates the following remarkable case of extensive division of the face by the cut of a sabre, and which was successfully treated by him. A Russian Colonel received from a French horseman "a stroke with a sabre, which cut off his nose at its base throughout its whole length. The instrument being directed obliquely, had effected a division of the two canine regions, and two lateral parts of the upper lip, extending into the substance of the two maxillary bones, on a level with the nasal fossæ. This division was bounded by the palatine arch, which formed a part of the flap, turned over upon the chin, and remaining adherent to the living parts of the face, merely by two small shreds of the upper lip, forming the commissures of the mouth. The entire extent of the nasal fossæ, and the cavity of the mouth, without the alveolar arch, were seen on one side, and on the other, the flap formed by the whole of the nose, the upper lip, and the palatine vault, hanging over the chin. One of my pupils, finding this flap cold, and attached to the other portions of the face, only at the two points, of which we have spoken, was proceeding to detach it entirely, and dress the wound, according to the indications it presented, when I arrived at the bedside of the patient. I laid aside the scissors of the surgeon, and after examining the wound, arranged every thing, for the purpose of employing the suture. I had some difficulty in removing the clots of blood which filled the nasal fossæ, and had been made hard by dust. I then detached the portion of the palatine vault, which adhered to the flap. It consisted of the anterior half of the superior alveolar arch. It had been separated from the remainder of the maxilla, on one side, between the canine and first molar, and on the other between the two molar teeth. I also detached from the flap, several pieces of the proper bones of the nose, and ascending process of the maxillary bones. The nose and lip were placed in their relative position, and I proceeded to reunite them with the surrounding parts, by the interrupted sature, commencing at the root of the nose, and descending successively on its sides, the edges of which were approximated by ten parallel points of the suture. A piece of fine linen dipped in salt water, was applied over the whole extent of the triangle, which was formed by the wound. I introduced into the nostrils two portions of large gum elastic sounds, for the purpose of preserving their form and diameter. They were commanded on the exterior by means of a thread, which I inserted into their anterior extremities. Graduated compresses were placed on the side of the nose, and a retentive bandage terminated the dressings. I had the satisfaction to learn, on my return from Moscow, that this superior officer had perfectly recovered without any deformity. The cure of this case is remarkable, on account of the seriousness of the wound, and the few vessels which kept up a communication between the flap, and the integuments of the face. Vitality was restored to the nose, and its reunion with the edges of the wound was exact and perfect.—*Am Jour. of Med. Sciences.*

16. *Deviation of the Menses.*—The following curious instance of anomalous menstruation, has been communicated to the Medical Society of Paris, by M. Bonfils, of Nantz, and is published in the *Transactions Medicales*, for October last. A girl of the town, twenty-one years of age, nervous temperament, had long been subject to hysteric spasms, especially just before menstruation. From the period when her menses first appeared, which was at the age of nine years, she menstruated regularly, the discharge continuing about eight days each period. Almost always, however, especially when she was chagrined, menstruation was accompanied by a sero-sanguinolent discharge, and often one of pure blood from the left mamma and axilla. In June, 1824, she was admitted into the "*Maison de Secours*," of Nantz, in the fifth month of pregnancy, with a syphilitic affection. She stated that during the first month of utero-gestation, she had a copious and continual uterine discharge, which weakened her much. During the following months, she menstruated as usual, and the flow was always accompanied with a sanguinolent oozing from the left axilla and mamma. She was delivered at the seventh month of a living child. The lochia appeared and followed the usual course, and after six weeks the patient was transferred to the venereal wards. She was then put under treatment for the venereal affection. On the 26th of Feb. 1825, her menses re-appeared, at the same time there was a discharge of blood from the parts already indicated, and which uninterruptedly continued until the 6th of the following March. During this period when these parts were wiped dry, in a few seconds the skin, which was of a natural color, was observed to become covered, for the size of a five franc piece, with a multitude of extremely small drops of blood, which, enlarging and uniting, formed in four or five minutes, two or three large drops, which, running together, flowed from the body. All the other functions were perfectly performed, and the patient did not apparently suffer from the discharge.

On the 7th of March, the sanguineous discharge from the vagina still continued abundant, but that from the axilla was replaced by another, which took place through the skin of the left flank, from the space of the size of a two franc piece. The patient had also a bloody taste in the mouth, and even expectorated some drops of that fluid. On the 8th and 9th, the discharge from the mamma continued, whilst that from the flank ceased, and another was established on the back, a little on the left, and towards the middle of the space, between the superior and internal angles of the scapulæ. The surface from which the discharge flowed, was two inches long, and one broad. The next day a discharge was established from a new place, viz. the epigastrium, the others continuing. The 12th, vaginal discharge considerably diminished, the flow from the the other parts, however, continued, and from the epigastrium became more abundant. The patient was leeched to the vulva, and the next day bled from the arm. On the 14th, the menstrual evacuation from the vagina increased, and the flow from the back and epigastrium ceased; that from the mamma con'inued. On the 15th, there appeared a slight oozing of blood from the lower and external portion of the left thigh. On the 16th, the menses had ceased towards midnight, and re-appeared at 3 o'clock the next afternoon. The discharges from the thigh and breast entirely ceased. The 17th, the menses were suppressed at 5 o'clock in the morning, and re-appeared at 3 o'clock in the afternoon as before. During the 18th, 19th, and 20th, there was a cessation of all the discharges. During the 21st, 22d, and 23d, the patient lost a few drops of blood from her left axilla, and during the night only, and the discharge appeared occasionally for the six succeeding days. The succeeding monthly evacuation was accompanied by only a small oozing from the left mamma, and which continued but eight days.

In August, 1826, this woman was again admitted into the "Maison de Secours," when she stated that for a year she had menstruated regularly and naturally, without having had any anomalous discharge. She was a third time admitted in June, 1827, for a syphilitic affection, when she stated that at the menstrual periods in April and May, her menses had been preceded, accompanied, and followed by a sanguinolent oozing from her axilla, the left mamma and flank; and in June, July, and August, the same circumstance occurred.—*Ib.*

17. *Anomalous Menstruation.*—The following case is recorded in the same Journal with the preceding, and was communicated to M. Bonfils, by M. Begin.—A young lady suffered suppression of her menses immediately after their first appearance in 1807, which was followed by swelling and suppuration of the glands of the neck. In 1815, she became affected with fluor albus, which was succeeded by an improvement in the health. In 1817, the leucorrhœa disappeared, and regularly afterwards, every month, the index finger of the left hand became tumefied, and covered with a violent tetter, from the surface of which, for three or four days, there oozed some drops of blood. This continued for three years, and the uterus resumed its menstrual functions, and the health of the patient was restored,—*Ib.*

18. *Constituent parts of Cinchona.*—According to Mr. R. Battley, the constituents of cinchona cordifolia, as determined by analysis, are, 1st, a free acid, easily disengaged by distilled water; 2d, quinine obtained by neutralizing the acid No. 1. with magnesia; 3d, quinine independent of that which is in combination with the acid No. 1.; 4th, bitter extractive; 5th, resin; 6th, gummy matter; 7th, gluten; 8th, tannin, combined with gallic acid; 9th, coloring matter; 10th, muriate of soda; 11th, sulphate of soda; 12th, iron; 13th, woody fibre.

Quinine is a bitter substance of the bark, forming characteristic salts with acids, and not containing any of the principles from No. 4 to 13; it appears to be an elementary substance.

In the preparation of the *liquor cinchonæ cordifoliæ*, it has been attempted to combine all the efficient properties of the bark, (excluding Nos. 6, 7, and 13, in which no active principle can be discovered,) and it is presumed that the object has been effected in that preparation.

The *sulphate of quinine* can only be partially efficient as a medicine, in consequence of the absence of all the properties above-mentioned, (from No. 4 to 13;) and the decided superiority of the *liquor cinchonæ* as a medicine, being fully admitted by many competent judges, it appears to me to be of importance to place that preparation under the attention of the profession at large.—*London Med. Gaz. December*, 1831.

19. *Lithotrity.*—About three years ago, M. Civiale had the gratification of relieving by this operation the venerable Dubois from a vesical calculus; and he has during the past winter achieved another triumph in the cure of M. Lisfranc, the celebrated surgeon of La Pitie. The cure is complete. The fact of two such men as Dubois and Lisfranc submitting to the operation of lithotrity in preference to lithotomy is a sufficient answer to the clamors of those who deny the utility of the former. That operation must now be admitted among the established resources of the art. That it will supercede lithotomy in all cases is not to be maintained; but that it is preferable in some seems now pretty well determined.—*Am. Jour. of Med. Sciences.*

20. *Rare Luxation of the Patella.*—Dr. Martin has communicated to the Medical Society of Lyons an instance of luxation of the right patella, the inner edge of which bone was in contact with the anterior and middle portion of the articular surface of the femur, its external edge projecting anteriorly. It occurred in a young lady fifteen years of age, whilst turning in her bed. Dr. M. reduced the luxation by flexing the thigh on the abdomen, and then seizing the patella with both hands, and drawing it outwards, turned it back to its natural position. The patella in this subject was small, and the ligaments somewhat relaxed.

Some surgeons have denied the possibility of such a luxation. Jean Sue, however, communicated to the Royal Academy of Surgery, in 1752, an instance of it, and another case is recorded by Dr. Wolff, in Rust's Magazine, for 1828. B. 27.—*Ib.*

21. *Ossification of the Pericardium.*—We find in our contemporary, the *London Medical and Surgical Journal,* for November last, an instance of this in a young man, twenty-seven years of age, who died of ascites, with peritoneal inflammation. The pericardium at its upper part, was completely ossified, forming a bony ring, enclosing the upper portion of the heart and extremities of the large vessels, an inch in breadth, and in some places a quarter of an inch in thickness. —*Am.Jour. of Med. Sciences.*

22. *Chrystallization of Perchloric Acid.*—M. Serullas states that the perchloric acid may be always crystallized by pursuing the following plan. Sulphuric and perchloric acid are to be successively introduced into a small retort, through a long tube; the beak of the retort is to be inserted into a tube curved and drawn to a fine point at the other extremity. Heat is to be applied, and when the liquid boils, and is kept in this state for some time, over a small fire, it will be seen to pass over into the tube and solidify there; the tube is to be kept cool with water; thick white fumes escape at the small end of it. The operation must be stopped before the mixture is discolored, and as soon as any liquid passes over which does not congeal. The experiment should always be performed with small quantities of the perchloric acid, say eight to ten drachms. Liquid perchloric acid may be concentrated by evaporation in a capsule, or what is better, in a small retort. The first portions that pass over are to be thrown away, as they are only water. M. Serullas has obtained it of the density of 1.65.—*Journ. de Chim. Med. and Journ. Phil. Col. Pharmacy,* 1832.

23. *Hydrocyanic Acid.*—M. Tilloy has succeeded in preparing a medical hydrocyanic acid, the effects of which do not vary, and which can be preserved for several years without any sensible alteration; this is

| | |
|---|---|
| Cyanide of mercury - - - - - | 1 part, |
| Distilled water - - - - - - | 4 parts, |
| Alcohol at 30° Baume - - - - | 4 parts. |

Dissolve the cyanide of mercury in the water, by aid of heat, and then mix it with the alcohol; add a very slight excess of hydro-sulphuric acid; throw in sub-carbonate of lead; agitate several times, and distil in a water bath, so as to obtain all the alcohol saturated with the hydrocyanic acid.—*Ibid.*

24. *Cyanide of Potassium.*—According to M. Chevallier, no work hitherto published contains an exact process for obtaining the cyanide of potash in a pure state. The usual plan is to calcine the ferro-hydrocyanate of potassa, then to dissolve it in distilled water, filter and evaporate to dryness. The procedure is impracticable, for the cyanide of potassa decomposes water on coming in contact with it.

The result therefore, would be hydrocyanate of potassa, and in heating this, all the hydrocyanic acid escapes, and the residue will be merely potassa.

I have prepared this salt by calcining the ferro-hydrocyanate of potassa, then separating the cyanide of potassa from the quadricarburet of iron by pure alcohol, on distilling which, cyanide of potassa is obtained very pure and white.—*Ibid.*

25. *Perchloric Acid as a test for the Mineral Alkalies.*—From a paper on this subject by M. Serullas, it appears, that if a few drops of perchloric acid be added to a solution of potash and soda, a precipitate of perchlorate of potash is instantly formed, the perchlorate of soda, or the soda, if there be not an excess of acid used, remains in solution, whence they can be separated by concentrated alcohol, which, at the same time precipitates the small quantity of perchlorate of potash held in solution. A solution of perchlorate of soda, on the addition of potash, lets fall, a precipitate of perchlorate of potash. When perchloric acid is added to solutions of sulphate, nitrate, hydrochlorate, bromate, hydrobromate or hydriodate of potash, it forms a precipitate, and the acids become free, and may be separated by means of concentrated alcohol. From these experiments it appears, that the perchloric acid forms an almost insoluble salt with potash, requiring to dissolve it more than sixty times its weight of water at 15 -|- 0. 2. That soda forms a very deliquescent salt, exceedingly soluble in water and concentrated alcohol. 3. That the perchlorate of potash, on the contrary, is insoluble in alcohol. Finally, that by means of perchloric acid, the salts of potash may be decomposed, and the acid separated.—*Ibid.*

